# Effects of Prey and Pollen Diets on the Reproduction and Longevity of *Orius insidiosus* (Say) (Hemiptera: Anthocoridae), a Key Thrips Predator

**DOI:** 10.3390/insects16111160

**Published:** 2025-11-13

**Authors:** Lovely Adhikary, Hugh Adam Smith, Sriyanka Lahiri

**Affiliations:** Department of Entomology and Nematology, Gulf Coast Research and Education Center, University of Florida, Wimauma, FL 33598, USA; hughasmith@ufl.edu (H.A.S.); lahiris@ufl.edu (S.L.)

**Keywords:** omnivory, supplemental diet, diet quality, biological control

## Abstract

*Orius insidiosus* (Say) (Hemiptera: Anthocoridae) is an important biological control agent for various soft-bodied arthropod pests. The effective mass rearing and reliable field performance of any biocontrol agent can depend on the quality of its diet. While a combination of insect prey and pollen has been tested previously, little information exists on how different prey and pollen types support survival and reproduction when provided alone. The current study evaluated adult longevity, reproductive span, and total and daily fecundity per female across five different diets. The first instar nymphs of *O. insidiosus* were reared on *Ephestia kuehniella* Zeller (Lepidoptera: Pyralidae) eggs + honey, pest thrips *Scirtothrips dorsalis* Hood (Thysanoptera: Thripidae) + honey, *Typha latifolia* L. (Poales: Typhaceae) pollen *+* honey, multifloral bee pollen + honey, and honey alone in laboratory conditions. The adults reared on *E. kuehniella* eggs + honey had the highest longevity, daily and total fecundity, and reproductive span, followed by those fed on *S. dorsalis* + honey. Two pollen diets supported reproduction and survival but were significantly less effective than insect-based diets; honey alone was insufficient to sustain reproduction. These results confirm the superior nutritional quality of *E. kuehniella* eggs for mass rearing while also showing that *O*. *insidiosus* can complete its lifecycle on pollen diets. Incorporating high-quality prey in the rearing system will improve colony productivity, whereas pollen supplements in the field may support predator persistence during periods of low pest density. This study contributes to optimizing both laboratory production and augmentation biological control programs using *O. insidiosus*.

## Simple Summary

*Orius insidiosus* (Say) (Hemiptera: Anthocoridae) is an important predator for thrips and other soft-bodied arthropods and is broadly used in biological control programs. Diet quality strongly influences the reproduction, longevity and overall performance of these predators both in mass rearing facilities and after augmentative release in the field. In this study, we evaluated the effects of different prey and pollen diets on the longevity and fecundity of *O. insidiosus*. The diets tested in the study were *Ephestia kuehniella* eggs, *Scirtothrips dorsalis* larvae, *Typha latifolia* and multifloral bee pollen and honey. Our results showed that *E. kuehniella* eggs supported the highest longevity and reproductive ability in adult *O. insidiosus,* making it the most suitable diet for efficient colony production. *S. dorsalis* also reinforced reproduction and demonstrated the potential of *O. insidiosus* as a biological control agent against this invasive thrips species. Although the pollen diets were less effective than insect-sourced diets, they enabled predators to complete their lifecycle. The findings of this study provide insights into improving both laboratory rearing and field performance of *O. insidiosus* in integrated pest management programs.

## 1. Introduction

Biological control agents are typically mass-produced in rearing facilities to ensure their ready availability in the field. Artificially supplied diets used in these facilities reduce rearing costs and may increase their potential as biological control agents [1]. Many predatory hemipterans are omnivores, able to feed on plants and other insect prey [2]. Omnivory in predatory insects may enhance development and survival by enabling them to acquire essential nutrients when animal prey is deficient [3].

In this context, *Orius* spp. (Heteroptera: Anthocoridae) are commercially important predators used in biological control programs, particularly for managing thrips in both open-field and greenhouse crop production [4,5]. In addition to thrips, they can also feed on other soft-bodied insects, including aphids (Hemiptera: Aphididae) [6] and whiteflies (Hemiptera: Aleyrodidae) [7]. *Orius* spp. are considered non-obligatory phytophagous insects as they can feed on plants [8], especially on xylem and mesophyll tissue, and consume water, sugar, and small amounts of amino acids [9]. They can also feed on pollen [10]. *Orius* spp. have been used in many biological control studies [11,12,13]. Their ability to survive on various diets may increase their reliability as biological control agents.

A previous study evaluated the fertility of *Orius majusculus* on two plant species, sweet alyssum (*Lobularia maritima* L.) (Brassicaceae) and green bean *Phaseolus vulgaris* L. (Fabaceae), with and without the supplement of *Ephestia kuehniella* Zeller (Lepidoptera: Pyralidae) eggs [14]. The authors [14] found that the *O. majusculus* exhibited higher fertility when the *Ephestia kuehniella* eggs were provided in addition to the host plants. Another study examined the fecundity and longevity of *Orius insidiosus* when fed on *Frankliniella occidentalis* (Pergande) (Thysanoptera: Thripidae) and multifloral pollen, including *Bidens pilosa* L. (Asteraceae), *Eucalyptus* spp. (Myrtaceae), *Citrus* spp. (Rutaceae), *Angycus* spp., and *Anagasta kuehniella* eggs as a diet, and found that the combination of pollen with insect prey resulted in the lowest pre-oviposition period in adults [15]. The life history parameters of *Orius laevigatus* (Fieber) and *Orius albidipennis* (Reuter) were studied under three dietary conditions: *E. kuehniella* eggs, multifloral pollen, and a combination of *E. kuehniella* and pollen [16]. *O*. *laevigatus* produced more eggs than *O. albidipennis*; however, when *E. kuehniella* eggs were provided with pollen, an increase in egg production was observed in *O. albidipennis* [16]. Therefore, the availability of plant nutritional sources, such as pollen, in addition to the insect diet, seems to enhance the biological parameters of *Orius* spp.

In other hemipteran predators, such as *Geocoris punctipes* (Say) (Hemiptera: Lygaeidae), a combined diet of lepidopteran eggs supplemented with a plant diet resulted in a shorter developmental time, increased nymphal survival rate, and higher body weight in newly emerged adults [17]. For the predatory stink bug, *Podisus maculiventris* (Say) (Hemiptera: Pentatomidae), supplementing the prey, Colorado potato beetle, *Leptinotarsa decemlineata* (Say) (Coleoptera: Chrysomelidae), with plant material increased the survival rate and reduced developmental time and the pre-ovipositional period [18].

Previous studies [15,16] have evaluated multifloral pollen as an alternate food source for *O. insidiosus*. However, very few studies [19] have used *Typha latifolia* L. (Typhaceae: Poales) pollen as a supplemental diet for rearing this species. *Typha* pollen is considered a nutritious alternative food for predators. For example, *Typha* pollen as supplemental food reduced the cannibalism among the predatory mite *Amblyseius herbicolus* Chant (Mesostigmata: Phytoseiidae) [20].

Another insect diet used in this study, *Scirtothrips dorsalis* Hood (Thysanoptera: Thripidae), is an invasive insect and had become a significant pest of many crops, including strawberries, *Fragaria x ananassa* Duchesne (Rosaceae), blueberries, *Vaccinium caesariense* (Ericaceae) [21], peppers, *Capsicum* sp. (Solanaceae) [22], and ornamentals [23] in the USA, after its establishment in 2005 [24]. *Scirtothrips dorsalis* is expanding its range to other southern and southeastern states of the USA [25]. The leading control option for this pest is insecticide application, which can lead to the development of insecticide resistance [26]. The biological control of *S. dorsalis* is heavily dependent on the release of predatory mites [27]. Since *Orius* spp. are well-known predators of thrips, *O. insidiosus* may have potential as a biological control agent for *S. dorsalis*. The inclusion of *S. dorsalis* as one of the diets in this study will further advance our understanding of whether *O. insidiosus* will successfully develop and reproduce by feeding on *S. dorsalis* and become a successful augmentation biological control agent for this pest.

As mentioned before, *O. insidiosus* is capable of feeding on different plant diets and may survive the prey scarcity period. Many studies have mentioned that *Orius* spp. feeding on pollen in the field [10,28] is an adaptive strategy [29] for biocontrol agents to maintain their population when prey density is low [16].

Mass rearing of predatory insects involves various advantages and disadvantages, including a nutritional regime that may increase body size but prolong the developmental time. Scientists must determine which is the most essential characteristic of a biocontrol agent for achieving successful control of the target pests. Additionally, the commercial rearing facilities must consider the cost of rearing these agents and regulate the nutritional sources to produce biological control agents that are economically sound and physically competitive [30], because an inferior diet can reduce nymphal survival and adult reproduction in predatory species [31].

By linking laboratory diet studies to mass rearing efficiency and field persistence, we aimed to provide practical insights to improve the use of *O. insidiosus* in thrips IPM programs. This study aims to evaluate the adult longevity and reproductive ability of *Orius insidiosus* females reared on two pollen diets, cattail pollen, *Typha latifolia* L. (Typhaceae), and multifloral bee pollen, and two insect diets, the eggs of the Mediterranean flour moth, *E. kuehniella* Zeller (Lepidoptera: Pyralidae), and chilli thrips, *Scirtothrips dorsalis,* with honey solution as a control. The null hypothesis of this experiment was that there would be no difference in adult longevity and reproductive ability of females reared on different diets. The alternative hypothesis was that the insect-based diets would result in increased longevity and reproductive ability than pollen and honey diets in adult *O. insidiosus* females.

## 2. Materials and Methods

All experiments were conducted at the Strawberry and Small Fruit Entomology lab at the University of Florida, Gulf Coast Research and Education Center (GCREC), Balm, FL (27.712490°, −82.302322°), at 25 ± 5 °C and 65 ± 5% RH, with a 16:8 L:D photoperiod. The diet treatments tested were as follows: (1) *Ephestia kuehniella* eggs and honey solution, (2) *Scirtothrips dorsalis* second instar larvae and honey solution, (3) cattail pollen *Typha latifolia* L. and honey solution, (4) multifloral bee pollen and honey solution, and (5) honey solution (control).

The *E. kuehniella* eggs were purchased from a commercial source (Beneficial Insectary, Redding, CA, USA). The *S. dorsalis* used in the experiment were from a colony maintained by the Strawberry and Small Fruit Entomology lab at GCREC on potted cotton plants, *Gossypium hirsutum* L. (Malvaceae) (MRC 270 Organic Cotton seeds; Brenham, TX, USA), with 5–6 leaves. The *S. dorsalis* colony was initially established in 2019 and was sourced from a naturally occurring field population in strawberry crops at the GCREC site. It was maintained in environmentally controlled growth rooms at 25 ± 5 °C and 60 ± 5% RH, with a 16:8 L:D photoperiod inside insect cages. The *T. latifolia* pollen and organic bee pollen were also purchased from commercial sources (Plum Dragon Herbs, Inc., Chester, MD, USA; Greenbow^®^, Cypress, CA, USA, respectively). Since the bee pollen used in this experiment was multifloral, information about the exact plant species in the pollen was not available. The honey used in the experiment was clover, *Trifolium repens* L. (Fabaceae) honey (Burleson Honey, Waxahachie, TX, USA), which was diluted to a 30% honey solution by mixing with de-ionized water.

The predator *O. insidiosus* was also acquired from a commercial source (BioBee Ltd., Sde Eliyahu, Israel). The adults of *O. insidiosus* were kept in lidded plastic containers (19.3 cm length × 28.26 cm width × 8.89 cm height) lined with tissue paper with a 5.08 cm × 5.08 cm vent on the lid. Organic sweet snap pea pods *Pisum sativum* L. (Fabaceae) were purchased, washed with tap water, air-dried, and then provided as an oviposition substrate. After 24 h, the pea pods with eggs were removed and placed into new, lidded containers. Wrinkled tissue paper was then placed inside the container to provide a hiding place and reduce the chance of cannibalism among the hatched nymphs. The eggs were evaluated every 24 h.

### 2.1. Rearing of O. insidiosus Nymphs

The 0 to 1-day-old *O. insidiosus* nymphs were removed from the containers using a fine brush and assigned to new containers with one of five different diets, as mentioned above (100 nymphs per diet type). Soup cups (12.7 cm in diameter) with screened lids, designed to facilitate ventilation, were used as the maturation cages.

At first, the tissue papers were placed at the bottom of the cups. Then the 0 to 1-day-old nymphs were transferred one by one into these cups on tissue paper with a fine brush. A small amount (0.15 g) of *E. kuehniella* eggs was provided as diet during initial instars. After the third instar, the amount of *Ephestia* eggs increased to 0.5 g. The quantity of *Ephestia* eggs was determined according to the rearing recommendations of Bueno et al. (2006) [32]. As the author suggested that *O. insidiosus* has a larger body size than other *Orius* spp. and that closed rearing conditions may increase the risk of cannibalism [32], prey amounts were increased to ensure that *Ephestia* eggs were not a limiting factor for the nymphs.

On the other hand, 1 g of cattail and bee pollen was sprinkled with the help of a thick brush inside the cup for even distribution of the food. Approximately 150–160 *S*. *dorsalis* second instar larvae were aspirated into a micro-pipette. The pipette was left open inside the cup to allow the prey to be released into the cup. The honey solution was given through a soaked cotton placed inside a piece of plastic drinking straw (1.5 cm in length). This was done to avoid soiling the floor of the maturation cage and to eliminate the chance of drowning of the nymphs. All the diet and the honey solution were changed every day to avoid mold development.

### 2.2. Experiment with Various Diets Using Adult O. insidiosus

After the nymphs matured into adults from each diet, a 24 h old pair (one male and one female) was placed in a smaller plastic cup with a 5.08 cm diameter. Males paired with each female remained in the rearing arena until death. Although a single mating event can be sufficient for oviposition in *O. insidiosus,* adult females can mate multiple times during their lifetime [33,34].

They were given the same diet on which they had been maintained since their first instar stage. Sweet snap pea pods were provided as oviposition substrate in these experimental cages. The oviposition substrate and the diet were changed every day, and the eggs laid on the pea pod were counted under the microscope (Stemi 508, Carl Zeiss, Oberkochen, Germany) at 40× magnification. Data collection included the oviposition per day per female (daily fecundity), the total number of eggs laid by a female, adult longevity, and length of reproductive period.

The experiment was repeated twice, referred to as trials 1 and 2. Trials 1 and 2 were performed at two different points in time and with new sets of insects, and followed the exact same experimental design. In trial 1, ten pairs of *O. insidiosus* were tested for each diet option, except for the honey (control) option, where four pairs were tested. In trial 2, ten pairs for *E. kuehniella* eggs + honey, *S. dorsalis* + honey, and cattail pollen + honey diets were tested, whereas in the case of bee pollen + honey, nine pairs were tested. Also, in trial 2, for the honey (control) option, five adult pairs were tested.

### 2.3. Data Analysis

Data was analyzed using the SAS OnDemand for Academics web platform (SAS Institute Inc., Cary, NC, USA). The diet treatments were considered as fixed effects, and the individual insects were modeled as a random effect in this experiment. The normality of the residuals was checked using the Shapiro–Wilk test (Proc Univariate). We used a completely randomized design, with four to ten replications per treatment as described above. The effects of different diets on the longevity and reproductive ability of adult *O. insidiosus* females were analyzed using a generalized linear mixed model (Proc GLIMMIX). The mean separation was performed using Tukey’s HSD at α = 0.05.

## 3. Results

Different diets significantly influenced total egg production in female *O. insidiosus* adults in both trials (F = 225.10; df = 4, 39; *p* < 0.0001, and F = 188.73; df = 4, 31.03; *p* < 0.0001, respectively). The superior quality of the *E*. *kuehniella* eggs + honey confirms the highest number of total eggs among all the treatments, followed by females fed on *S. dorsalis* + honey. The number of eggs produced by individuals reared on cattail pollen and honey, as well as multifloral bee pollen and honey, did not differ significantly from each other. The total eggs produced in the honey-only treatment were the lowest (Table 1).

A similar trend was observed in the daily oviposition experiments in both trials (F = 132.14; df = 4, 39; *p* < 0.0001 and F = 74.36; df = 4, 31.14; *p* < 0.0001, respectively). Oviposition per day per female was highest in the adults reared on *E. kuehniella* eggs + honey, followed by adults reared on *S. dorsalis* + honey diets. The number of eggs produced per female per day did not differ significantly between the two pollen + honey diets (Table 2).

The longevity of adults also differed significantly among different diets in both trials (F = 121.02; df = 4, 39; *p* < 0.0001, and F = 116.76; df = 4, 31.46; *p* < 0.0001). *E. kuehniella* eggs + honey were found to be the finest diet to support longevity in adult *O. insidiosus*. Individuals fed the *S. dorsalis* + honey diet lived longer than those fed the two other pollen + honey diets and the honey-only diet (Table 3).

Both insect + honey diets resulted in similar length of reproductive periods (F = 80.37; df = 4, 39; *p* < 0.0001 and F = 102.33; df = 4, 39; *p* < 0.0001) in *O. insidiosus* adults. The reproductive period of female *O. insidiosus* fed on the pollen + honey diets was significantly lower than the insect + honey diets. However, both pollen diets supported the same reproductive length in both trials (Table 4). The honey-only diet resulted in the shortest reproductive period.

## 4. Discussion

In the current study, diet significantly affected the longevity and reproductive ability of *O. insidiosus*. *Ephestia kuehniella* eggs + honey solution was the most effective diet for daily and total fecundity, longevity, and reproductive period in adult *O. insidiosus,* followed by *S. dorsalis* + honey. On the other hand, the pollen diets, cattail pollen and bee pollen, did not differ from each other and supported the same level of reproduction and longevity, but were less effective than the insect diets. The individuals fed on honey only performed poorly, pointing to the fact that a carbohydrate-rich diet alone cannot boost the lifecycle parameters of *O. insidiosus*.

The possible reason for this observation is that the lepidopteran eggs are richer in nutritional quality compared to other insect diets [35,36]. These eggs are also less challenging to handle than highly mobile, cryptic prey such as *S. dorsalis*. *Orius* spp. has demonstrated that high-quality protein-rich insect diets, particularly *E. kuehniella* eggs, better support oviposition, longer lifespan, longer reproductive time, and shorter developmental period compared with pollen or other factitious diets [15,29]. Supplemental pollen can sometimes enhance reproductive success, but the effects vary among species and dietary combinations [15,16,29,37]. Our results align with the findings of Tommasini et al. (2004) [38], where the oviposition and longevity of the adult *Orius* spp. were superior when the *E. kuehniella* eggs were provided compared to *F. occidentalis*.

The total egg production and eggs per day per female were significantly lower in pollen diets than in the insect diets in our study. The lower protein content of the pollen diet can hamper egg maturation in females [8]. Between the two pollen diets tested in our study, *T. latifolia* contains 17 percent protein [39]. Bee pollen contains 21.3 percent protein [40]. However, egg production, longevity, and reproductive duration did not differ between these two pollen diets in our study.

Although the biochemical quantification of macronutrients in the diet was beyond the scope of this study, the nutritional composition is important for reproduction and longevity in insects. *Ephestia kuehniella* eggs are known to be rich in protein and contain balanced proportions of essential amino acids and lipids that support reproduction and longevity in predatory insects [41,42]. In contrast, pollen diets such as cattail and multifloral bee pollen generally contain lower crude protein levels and higher carbohydrate fractions [39,40]. These nutritional differences likely explain the superior fecundity and longevity observed when *O. insidiosus* females were fed insect-based diets compared to pollen. Our findings suggest that protein-rich diets provide the amino acids and lipids required for egg production and extended adult longevity in *O. insidiosus*. As nymphal survival was not evaluated in this study, we cannot conclude that these plant-based diets are effective throughout the lifecycle of these predators or suitable for mass rearing facilities. Instead, our results suggest that pollen and honey may function as supplemental resources rather than primary developmental diets. In a natural environment where prey density fluctuates, the availability of floral resources may help maintain predator populations until prey becomes abundant again.

Apart from diet, longevity can also influence insect oviposition [43]. The longevity of the *O. insidiosus* females ranged from 50.9 days when fed on *Ephestia* eggs to 40.5 days when fed on pollen [16]. In another study with *O. laevigatus*, the longevity was 50.2 days for adults feeding on *Ephestia* eggs when reared at 23 °C and 70 ± 5% RH [44]. In our study, adult longevity was significantly lower than that reported in previous studies, ranging from 29.6 to 31.4 days across two trials. This difference could be attributed to variations in ambient temperature during rearing [45]. The authors reported lower longevity of 24.83 to 26.44 days when reared in 27 ± 1 °C and 60 ± 10% RH [45].

In the current study, the total number of eggs laid by females reared on *Ephestia* and honey was 141.3 and 123.5 in Trial 1 and Trial 2, respectively. (Table 1). Another study reported much higher fecundity in *O. insidiosus* females (190.3 eggs per female) when *Ephestia* eggs were offered as food [15]. In studies with *Orius laevigatus,* the total fecundity fluctuated from 109.8 to 150.4 when reared on *Ephestia* eggs [16,44]. These differences may be due to variations in ambient temperatures and different oviposition substrates used in the experiments. In the current study, sweet snap peas were used, whereas Calixto et al. (2013) [15] used stems of *Bidens pilosa* L. (Asterales: Asteraceae) as an oviposition substrate. Different oviposition substrates can also lead to differences in the number of eggs laid by females [46]. Larval diet can also influence insect reproductive ability [47]. However, others argue that the adult diet has more effect on reproduction than the nymphal diet [44]. In this study, insects were fed the same diet throughout their lives.

A previous study showed that *O. insidiosus* nymphs fed exclusively on bee pollen had lower survival than those fed a diet supplemented with *E. kuehniella* eggs [48]. In our study, we examined the effects of different diets combined with a honey solution. Bee pollen resulted in significantly lower longevity and reproductive ability compared to *E. kuehniella* and *S. dorsalis. Orius insidiosus* was barely able to produce eggs when reared on a honey solution alone, and the longevity and reproductive ability of the adults were severely reduced. In our study, only 4–5 pairs of newly emerged adults developed from the honey solution diet, even though 100 nymphs (freshly hatched) were initially used to obtain adults, similar to the case with the other diet options. There was also a high level of cannibalism among the nymphs and adults, who were provided with only the honey solution. The individuals with pollen and honey had also experienced cannibalism, but at a lower level, possibly because pollen serves as an alternative food with some protein availability, which reduces the chance of cannibalism [49]. A dietary source with poor nutritional quality (honey) can lead to higher cannibalism in predators [50]. The carbohydrate in the honey diet can be a source of energy [51] and acts as a feeding stimulant for insects [52]. However, the honey diet was insufficient as a protein-rich diet, which is crucial for the growth and reproduction of predatory insects. A nutrient-rich diet can significantly increase body weight [53] and egg production [30].

Diet studies are fundamental for the successful mass rearing of biocontrol agents used in augmentation biocontrol. The fitness of natural enemies is directly related to the quality of food they were provided with during rearing, which determines the survival, reproductive ability, and overall performance after release.

This study provides the first experimental evidence that *Scirtothrips dorsalis* can serve as suitable prey for *O. insidiosus*, confirming its potential role as a biological control agent against this invasive pest. Previous work with *O. insidiosus* primarily focused on *Frankliniella* spp. [4,54], among the pest thrips. Our results show that *O. insidiosus* completed its lifecycle and successfully reproduced when fed on *S. dorsalis.* This information suggests that *S. dorsalis* is acceptable and adequate prey for *O. insidiosus*.

## 5. Conclusions

Among the diets tested, *E. kuehniella* eggs supported the highest fecundity, longevity, and reproductive span, underscoring their value for mass rearing facilities. These findings can improve the efficiency of the mass rearing programs. Although the production costs associated with *E*. *kuehniella* eggs are relatively high, their superior nutritional quality ensures greater colony productivity and availability of robust predators for augmentation releases. The inclusion of *S. dorsalis* as prey in the study revealed that *O*. *insidiosus* can successfully develop and reproduce on this invasive pest, supporting its potential role in the integrated management of thrips in strawberries and other essential crops. Given that *S. dorsalis* is becoming resistant to commercial broad-spectrum insecticides, the use of *O*. *insidiosus* could provide a sustainable alternative to chemical applications within IPM programs.

Although pollen diets were less effective than insect prey, *O*. *insidiosus* completed its lifecycle on both *Typha* and multifloral bee pollen. The results suggest that pollen availability in crop landscapes, whether through banker plants or supplemental pollen application, can sustain predator populations during periods of low prey density. By enhancing persistence and establishment, pollen supplementation may improve the effectiveness of *O. insidiosus* following release.

From an applied perspective, these findings contribute to both laboratory and field applications of *O. insidiosus*. Optimizing rearing diets with *E. kuehniella* eggs, reliable production of high-quality predators while provisioning pollen in crop environments can maintain populations between pest outbreaks. Together, these strategies strengthen the integration of *O. insidiosus* into IPM programs.

## Figures and Tables

**Table 1:** Mean (±SE) total fecundity of *Orius insidiosus* adult females reared on different diets. N = number of replicates. For most diets, N = 10 females per trial; Bee pollen: N = 10 (Trial 1), 9 (Trial 2); Honey solution: N = 4 (Trial 1), 5 (Trial 2). *p*-values of overall ANOVA obtained by Proc GLIMMIX (SAS OnDemand for Academics). Different letters indicate a significant difference among diets (*p* < 0.05, post hoc Tukey’s HSD test).

Diet	Trial 1		Trial 2	
	Replication	Fecundity	Replication	Fecundity
*E. kuehniella* eggs + honey	10	141.3 ± 5.69 A	10	123.5 ± 5.84 A
*S. dorsalis* + honey	10	62.8 ± 2.78 B	10	78.1 ± 3.19 B
Cattail pollen + honey	10	35.9 ± 1.99 C	10	40.8 ± 1.31 C
Bee pollen+ honey	10	29.8 ± 1.23 C	9	28.00 ± 1.82 C
Honey	4	1.00 ± 0.41 D	5	0.8 ± 0.49 D

**Table 2:** Mean (±SE) oviposition per day per female of *Orius insidiosus* adult reared on different diets. N = number of replicates. For most diets, N = 10 females per trial; Bee pollen: N = 10 (Trial 1), 9 (Trial 2); Honey solution: N = 4 (Trial 1), 5 (Trial 2). *p*-values of overall ANOVA obtained by Proc GLIMMIX (SAS OnDemand for Academics). Different letters indicate a significant difference among diets (*p* < 0.05, post hoc Tukey’s HSD test).

Diet	Trial 1		Trial 2	
	Replication	Daily Oviposition	Replication	Daily Oviposition
*E. kuehniella* eggs + honey	10	5.75 ± 0.30 A	10	5.17 ± 0.28 A
*S. dorsalis* + honey	10	2.88 ± 0.07 B	10	3.54 ± 0.23 B
Cattail pollen + honey	10	1.83 ± 0.09 C	10	2.65 ± 0.15 C
Bee pollen+ honey	10	1.53 ± 0.06 C	9	1.92 ± 0.07 C
Honey	4	0.42 ± 0.14 D	5	0.26 ± 0.16 D

**Table 3:** Mean (±SE) adult longevity in days of *Orius insidiosus* females reared on different diets. N = number of replicates. For most diets, N = 10 females per trial; Bee pollen: N = 10 (Trial 1), 9 (Trial 2); Honey solution: N = 4 (Trial 1), 5 (Trial 2). *p*-values of overall ANOVA obtained by Proc GLIMMIX (SAS OnDemand for Academics). Different letters indicate a significant difference among diets (*p* < 0.05, post hoc Tukey’s HSD test).

Diet	Trial 1		Trial 2	
	Replication	Adult Longevity (Days)	Replication	Adult Longevity (Days)
*E. kuehniella* eggs + honey	10	29.6 ± 0.73 A	10	31.4 ± 1.11 A
*S. dorsalis* + honey	10	25.4 ± 0.69 B	10	26.2 ± 0.88 B
Cattail pollen + honey	10	21.6 ± 0.79 C	10	18.2 ± 0.66 C
Bee pollen+ honey	10	21.0 ± 0.62 C	9	17.11 ± 0.81 C
Honey	4	2.25 ± 0.25 D	5	2.4 ± 0.88 D

**Table 4:** Mean (±SE) of the length of reproductive period of *Orius insidiosus* adult females reared on different diets. N = number of replicates. For most diets, N = 10 females per trial; Bee pollen: N = 10 (Trial 1), 9 (Trial 2); Honey solution: N = 4 (Trial 1), 5 (Trial 2). *p*-values of overall ANOVA obtained by Proc GLIMMIX (SAS OnDemand for Academics). Different letters indicate a significant difference among diets (*p* < 0.05, post hoc Tukey’s HSD test).

Diet	Trial 1		Trial 2	
	Replication	Reproductive Period (Days)	Replication	Reproductive Period (Days)
*E. kuehniella* eggs + honey	10	24.8 ± 0.771 A	10	24 ± 0.73 A
*S. dorsalis* + honey	10	22 ± 0.774 AB	10	21.6 ± 0.81 A
Cattail pollen + honey	10	19.7 ± 0.76 B	10	15.3 ± 0.57 B
Bee pollen+ honey	10	19.3 ± 0.71 B	9	14.77 ± 0.878 B
Honey	4	1.00 ± 0.408 C	5	0.8 ± 0.489 C

## Data Availability

The original contributions presented in this study are included in the article. Further inquiries can be directed to the corresponding author.
